# D-mannose blocks S-adenosylmethionine generation to suppress macrophage IL-1β expression

**DOI:** 10.3389/fimmu.2026.1895029

**Published:** 2026-08-21

**Authors:** Yingyi Chen, Junji Xu, Jingfei Fu, Juan Du, Ziqing Feng, Minfeng Wang, Yi Liu, Yitong Liu

**Affiliations:** 1 Laboratory of Tissue Regeneration and Immunology and Department of Periodontics, Beijing Key Laboratory of Tooth Regeneration and Function Reconstruction, School of Stomatology, Capital Medical University, Beijing, China; 2 College of Stomatology, Chongqing Key Laboratory of Oral Diseases and Biomedical Sciences, Chongqing Municipal Key Laboratory of Oral Biomedical Engineering of Higher Education, Chongqing Medical University, Chongqing, China; 3 Department of Neurosciences, School of Medicine, Case Western Reserve University, Cleveland, OH, United States

**Keywords:** D-mannose, Histone Methylation, Macrophages, One-carbon metabolism, SAM

## Abstract

**Background and aims:**

Inflammatory stimuli drive metabolic reprogramming in macrophages, supplying energy, reducing equivalents, and methyl donors required for the production of inflammatory mediators such as IL-1β; however, metabolic strategies to regulate this pathway remain unclear. Here, we aimed to define the mechanism by which D-mannose suppresses one-carbon metabolism in inflammatory macrophages and thereby epigenetically restrains inflammatory responses.

**Methods:**

Untargeted metabolomics was used to define the global metabolic effects of D-mannose in inflammatory macrophages. 6-phosphogluconate dehydrogenase activity and real-time ATP rate assays were performed to assess nicotinamide adenine dinucleotide phosphate (NADPH) and adenosine triphosphate (ATP) generation. S-adenosylmethionine (SAM) and cytokine levels were measured by enzyme-linked immunosorbent assay, and epigenetic regulation at the *Il1b* promoter was examined by chromatin immunoprecipitation-qPCR and immunoblotting. A murine full-thickness skin wound model with macrophage depletion was used for *in vivo* validation.

**Results:**

D-mannose suppressed metabolic flux through glycolysis, the tricarboxylic acid (TCA) cycle, and the pentose phosphate pathway, resulting in reduced ATP and NADPH production. This metabolic restriction impaired one-carbon metabolism, decreased SAM abundance, and reduced histone H3 lysine 36 trimethylation enrichment at the *Il1b* promoter, thereby repressing *Il1b* transcription. These effects were reversed by exogenous SAM supplementation. Macrophages exhibited a metabolic bottleneck associated with low mannose phosphate isomerase (*Mpi*) expression. Consistently, *Mpi* overexpression reversed the D-mannose-induced reductions in ATP and SAM levels, as well as *Il1b* expression. Topical D-mannose also lowered SAM and IL-1β levels in wound tissues and accelerated wound healing *in vivo*.

**Conclusions:**

D-mannose suppresses macrophage pro-inflammatory transcription through a one-carbon metabolism-epigenetic axis, supporting its potential as a metabolism-targeted strategy for inflammatory wound repair.

## 1 Introduction

Metabolic reprogramming is a key mechanism underlying phenotypic and functional transitions in macrophages responding to microenvironmental stimuli (1). LPS/Toll-like receptor (TLR) stimulation is typically accompanied by upregulated glycolytic flux and TCA cycle disruption. Key glycolytic enzymes, including hexokinase 1 (HK1) and pyruvate kinase M2 (PKM2), influence NLRP3 inflammasome activation and enhance the maturation of IL-1β and IL-18 (2, 3). Succinate accumulated in the disrupted TCA cycle promotes IL-1β production via HIF-1α (4). Meanwhile, the PPP, a glycolytic branch, supports inflammatory responses by supplying NADPH (5). Collectively, these carbon metabolic networks—centered on glycolysis, the TCA cycle, and the PPP—constitute the essential metabolic and energetic foundation for inflammatory macrophages to sustain their effector state.

Carbon metabolism is tightly coupled to one-carbon metabolism. One-carbon metabolism comprises the folate and methionine cycles, and its flux is sustained by metabolic inputs including ATP and NADPH (6, 7). In the folate cycle, methylenetetrahydrofolate reductase (MTHFR) utilizes NADPH as a reducing equivalent to convert 5,10-methylenetetrahydrofolate (5,10-mTHF) to 5-methyltetrahydrofolate (5-mTHF) (8). Subsequently, 5-mTHF serves as a methyl donor to remethylate homocysteine to methionine. In the methionine cycle, methionine is converted to SAM in an ATP-dependent manner; SAM then functions as a universal methyl donor supporting epigenetic modifications such as histone methylation, thereby modulating chromatin accessibility and gene transcription (9). Studies have demonstrated that one-carbon metabolism drives IL-1β production in inflammatory macrophages by generating SAM to support histone H3K36 methylation (6). Accordingly, the functional coupling between carbon metabolism and one-carbon metabolism contributes to macrophage inflammatory responses, yet the metabolic regulatory strategies targeting this pathway remain unclear.

D-mannose is a naturally occurring C-2 epimer of glucose that enters cells primarily via hexose transporters on the plasma membrane (10). Under supraphysiological conditions, intracellular D-mannose is phosphorylated to Man-6-P, and its subsequent metabolism is rate-limited by *Mpi* expression. In cells with relatively low MPI levels, Man-6-P tends to accumulate and feedback-inhibit upstream hexose metabolism (10, 11). Torretta et al. (12) demonstrated that D-mannose competitively inhibits glucose metabolism in pro-inflammatory macrophages, blocking the accumulation of the TCA cycle breakpoint metabolite succinate and thereby downregulating HIF-1α-driven IL-1β expression. However, carbon metabolism constitutes a coupled network of multiple pathways, such as glycolysis, the TCA cycle, and the pentose phosphate pathway. Whether the upstream hexose metabolic blockade induced by D-mannose disrupts this network and consequently impairs one-carbon metabolism has yet to be systematically elucidated.

This study aimed to investigate the mechanistic basis by which D-mannose suppresses one-carbon metabolism in inflammatory macrophages to exert epigenetic regulation. We found that, due to the “metabolic bottleneck” imposed by low *Mpi* expression in macrophages, D-mannose not only inhibited glycolysis and the TCA cycle but also markedly constrained the PPP, leading to a concurrent reduction in intracellular ATP and NADPH levels. This consequently impaired one-carbon metabolism and diminished the SAM pool. SAM insufficiency further attenuated the enrichment of the activating histone mark H3K36me3 at the *Il1b* promoter, thereby epigenetically suppressing macrophage *Il1b* expression. *In vivo*, topical application of D-mannose gel lowered SAM and IL-1β levels in wound tissues and accelerated full-thickness skin wound healing in a macrophage-dependent manner. Overall, this work provides a mechanistic foundation for D-mannose as a metabolism-targeted epigenetic intervention in wound repair.

## 2 Materials and methods

### 2.1 Reagents and antibodies

Detailed information on the antibodies and reagent kits used in this study is provided in **Supplementary Table 1**.

### 2.2 Animals

Male C57BL/6 mice (6–8 weeks old) were purchased from SPF Biotechnology Co., Ltd. (Beijing, China). Animals were housed in a specific pathogen-free (SPF) facility with *ad libitum* access to standard chow and purified water. All animal experiments were performed in accordance with the ARRIVE (Animal Research: Reporting of *In Vivo* Experiments) guidelines and the National Institutes of Health (NIH) Guide for the Care and Use of Laboratory Animals. The study protocols were approved by the Ethics Committee of Beijing Stomatological Hospital, Capital Medical University (Approval No. KQYY-202206-007).

### 2.3 Isolation and culture of mouse peritoneal macrophages

Mouse peritoneal macrophages were elicited by intraperitoneal injection of 4% thioglycolate broth (Sigma-Aldrich, St. Louis, MO, USA). Cells were harvested by peritoneal lavage 3–5 days post-injection. Primary macrophages were seeded at a density of 2 × 10^6^ cells per well in 12-well plates and cultured in DMEM (Invitrogen, Carlsbad, CA, USA) supplemented with 10% heat-inactivated fetal bovine serum (FBS; Equitech-Bio, Kerrville, TX, USA), 100 U/mL penicillin, 100 μg/mL streptomycin (Invitrogen), and 2 mM L-glutamine (Invitrogen). Cells were assigned to the following experimental groups: (1) glucose-free DMEM + 1 μg/mL LPS (InvivoGen, San Diego, CA, USA); (2) D-glucose (25 mM) DMEM + 1 μg/mL LPS; and (3) D-mannose (25 mM) DMEM + 1 μg/mL LPS.

### 2.4 Untargeted metabolomics

Cell samples from each group (initially n = 6; 1 × 10^7^ cells per sample) were subjected to untargeted metabolomics analysis. Metabolites were extracted with pre-chilled 80% methanol. After vortexing and incubation on ice, the samples were centrifuged at 14,000 × g for 15 min at 4 °C, and the supernatants were collected for liquid chromatography–tandem mass spectrometry (LC-MS/MS) analysis on a Thermo Q Exactive HF-X mass spectrometer coupled with an Ultimate 3000 UHPLC system (Thermo Fisher Scientific, Waltham, MA, USA). Chromatographic separation was performed in negative-ion mode on an HSS T3 column (1.8 μm, 2.1 × 100 mm; Waters, Milford, MA, USA) with a gradient elution of 0.1% formic acid in water (mobile phase A) and 0.1% formic acid in acetonitrile (mobile phase B). Mass spectrometric detection was carried out using a heated electrospray ionization (HESI) source in negative-ion mode, with full MS scanning followed by data-dependent acquisition. Raw data were processed using Compound Discoverer software, and metabolite annotation was performed by matching against the KEGG, METLIN, HMDB, and LIPIDMaps databases. Following screening by principal component analysis with a 95% confidence ellipse, globally outlying samples were excluded from the dataset. Consequently, orthogonal partial least squares discriminant analysis (OPLS-DA), differential metabolite analysis, and KEGG pathway enrichment analysis were performed using n = 5 per group. For individual metabolite abundance analyses, data points without evidence of sample-specific outlier behavior were retained. The corresponding raw data are provided in **Supplementary Tables 2**–**S4**.

### 2.5 6-phosphogluconate dehydrogenase activity assay

Peritoneal macrophages were collected, and 6PGD activity was measured using a 6PGD Activity Assay Kit (Solarbio, Beijing, China) according to the manufacturer’s instructions. Briefly, cells were resuspended in extraction buffer at a ratio of 1 mL per 5 × 10^6^ cells, sonicated, and centrifuged at 8,000 × g for 10 min at 4 °C to collect the supernatant. In this assay, 6PGD catalyzes the conversion of NADP^+^ to NADPH in the pentose phosphate pathway. Because NADPH, but not NADP^+^, shows a characteristic absorbance at 340 nm, 6PGD activity was determined by monitoring the increase in absorbance at 340 nm over 5 min. Enzymatic activity was calculated based on the rate of NADPH generation and normalized to protein concentration (U/mg protein).

### 2.6 Real-time ATP rate assay using the seahorse platform

Mouse peritoneal macrophages were seeded in Seahorse XFe24 Cell Culture Microplates at a density of 8 × 10^4^ cells/well and cultured at 37 °C in 5% CO_2_. After cell adhesion, the medium was replaced with the following conditions: (1) glucose-free DMEM; (2) glucose-free DMEM + 1 μg/mL LPS; (3) D-glucose (25 mM) DMEM + 1 μg/mL LPS; and (4) D-mannose (25 mM) DMEM + 1 μg/mL LPS. Cells were incubated for 24 h at 37 °C in 5% CO_2_. The Real-Time ATP Rate Assay was performed according to the manufacturer’s instructions, and basal oxygen consumption rate (OCR) and extracellular acidification rate (ECAR) were recorded. Following sequential injection of oligomycin and rotenone/antimycin A, OCR, ECAR, and proton efflux rate (PER) values were used to calculate mitoATP and glycoATP production rates using the Seahorse XF Report Generator.

### 2.7 ATP level assay

Cells were washed twice with cold PBS and lysed. The supernatant was collected after centrifugation at 12,000 × g for 5 min at 4 °C, and ATP levels were quantified using an ATP Assay Kit (Beyotime, Shanghai, China). Briefly, 100 μL of ATP detection working solution was added to each well of the assay plate, followed by rapid addition and mixing of the sample or standard solution. After a 2-s equilibration, relative luminescence units were measured using a chemiluminescence reader. ATP levels were normalized to protein concentration (nmol/mg protein).

### 2.8 Enzyme-linked immunosorbent assay

Wound tissues were homogenized using a mechanical homogenizer and centrifuged at 2,000–3,000 rpm for 20 min. The supernatant was collected as wound tissue lysate. Protein concentrations were determined using the DC Protein Assay Kit (Bio-Rad, Hercules, CA, USA), and all results were normalized to protein concentration. ELISA kits for IL-1β, TNF-α, and IL-6 (BioLegend, San Diego, CA, USA) and SAM (mlbio, Shanghai, China) were used to quantify target analyte levels in wound tissue lysate, serum, cell culture supernatant, and cell lysate. All procedures were performed strictly according to the manufacturers’ instructions.

### 2.9 Western blot

Cell and skin tissue samples were washed twice with cold PBS and lysed in RIPA buffer (Beyotime, Shanghai, China) supplemented with protease and phosphatase inhibitors. After protein quantification, equal amounts of protein were loaded onto 7.5% SDS-polyacrylamide gels and transferred onto Immobilon™-P PVDF membranes (Millipore, Billerica, MA, USA). Membranes were blocked with 5% non-fat milk and incubated overnight at 4 °C with the following primary antibodies: anti-trimethyl-histone H3 (Lys27) (C36B11), anti-trimethyl-histone H3 (Lys4) (C42D8), anti-histone H3 (D1H2), and anti-β-actin (8H10D10) (1:1,000; Cell Signaling Technology, Danvers, MA, USA), as well as anti-IL-1β and anti-histone H3 (trimethyl K36) (1:1,000; Abcam, Cambridge, UK). After washing, membranes were incubated with HRP-conjugated secondary antibodies (1:2,000; Pierce, Rockford, IL, USA). Protein bands were visualized using an enhanced chemiluminescence (ECL) kit (Thermo Fisher Scientific, Waltham, MA, USA). Densitometric analysis of the bands was performed using ImageJ and normalized to Histone H3 or β-actin.

### 2.10 Chromatin immunoprecipitation and ChIP-qPCR

Cells were seeded into 100-mm culture dishes at a density of 1 × 10^7^ cells/dish. Following the indicated treatments, chromatin immunoprecipitation (ChIP) was performed using a ChIP Assay Kit (Beyotime) according to the manufacturer’s protocol. Immunoprecipitated DNA was analyzed by real-time PCR (ChIP-qPCR). Primer sequences are listed in **Supplementary Table 5**.

### 2.11 RNA extraction and real-time PCR

Total RNA was extracted from wound tissues and cultured cells using the Ultrapure RNA Kit (CWBIO, Suzhou, China) according to the manufacturer’s instructions. Complementary DNA was synthesized from 1 μg of total RNA using a Reverse Transcription Kit (Takara Bio Inc., Shiga, Japan). Real-time PCR was performed using TB Green Premix Ex Taq II (Tli RNaseH Plus) (Takara). Primer sequences are listed in **Supplementary Table 5**. Relative gene expression was calculated using the 2^−ΔΔCt^ method.

### 2.12 Flow cytometry

Macrophages were harvested, washed, and resuspended in PBS containing 5% BSA to obtain a single-cell suspension. For intracellular cytokine staining, cells were stimulated with a cell activation cocktail for 4 h at 37 °C. Dead cells were excluded using the Zombie Yellow™ Fixable Viability Kit (BioLegend). Fc receptors were blocked with anti-CD16/32 antibody (BioLegend). Surface staining was performed using fluorochrome-conjugated antibodies against F4/80 (BM8)-BV421 and CD11b (M1/70)-FITC (BioLegend). Cells were then fixed and permeabilized using the Fixation/Permeabilization Buffer Set (eBioscience, San Diego, CA, USA), followed by intracellular staining with anti-IL-1β (pro-form) (NJTEN3)-APC (eBioscience). Data were acquired on a BD FACSCanto™ flow cytometer (BD Biosciences, San Jose, CA, USA) and analyzed using FlowJo software (TreeStar, Ashland, OR, USA).

### 2.13 Lentiviral transduction

Macrophages were seeded in 6-well plates at a density of 1 × 10^6^ cells/well. After removing the culture medium and washing twice with PBS, 1 mL of fresh medium containing 1× transfection enhancer was added. Control or *Mpi*-overexpressing lentiviral vectors were added at a multiplicity of infection of 50, and cells were incubated for 16 h. At 48 h post-transduction, stably transduced cells were selected with 1 μg/mL puromycin (Sigma-Aldrich) for 72 h. *Mpi*-overexpressing cells were verified and used for subsequent experiments.

### 2.14 Full-thickness skin wound model

Male C57BL/6 mice were randomly assigned to control and D-mannose groups (n = 15 per group). The dorsal skin was shaved, and a single circular full-thickness wound (5 mm in diameter) was created following routine disinfection under general anesthesia. Briefly, mice were anesthetized by intraperitoneal injection of sodium pentobarbital at a dose of 50 mg/kg. A 2% (w/v) methylcellulose gel containing either 1.1 M D-mannose or PBS (vehicle control) was freshly prepared. Wounds in the D-mannose group were treated daily with topical application of D-mannose gel, while the control group received vehicle gel. Wound area was measured on postoperative days 0, 1, 3, 5, 7, and 9, and quantified using ImageJ software (National Institutes of Health, Bethesda, MD, USA). The percentage of unhealed wound area was calculated relative to the initial wound area at day 0. Mice from each group were euthanized on postoperative days 3, 5, and 7 by gradual-fill carbon dioxide inhalation, followed by cervical dislocation to ensure death. Wound tissues were then harvested for subsequent analyses.

### 2.15 Macrophage depletion with clodronate liposomes

To achieve macrophage depletion *in vivo*, clodronate liposomes (Formumax, Sunnyvale, CA, USA) were administered as previously described (13). One day prior to wounding, 150 μL of clodronate liposomes (7 mg/mL) or control liposomes were injected intraperitoneally. A second intraperitoneal injection of 100 μL of clodronate liposomes (7 mg/mL) or control liposomes was administered on day 1 post-wounding. Wound area was measured on days 0 and 3 post-injury, and wound healing rates were calculated. Mice were euthanized on day 3 post-wounding, and wound tissues were harvested for subsequent analyses.

### 2.16 Histological analysis

Wound tissues were fixed in 4% paraformaldehyde, dehydrated, and embedded in paraffin. Serial sections (5 μm) were prepared and stained with hematoxylin and eosin (H&E). Collagen deposition was assessed by Masson’s trichrome staining using a commercial kit (Solarbio) according to the manufacturer’s instructions.

### 2.17 Immunohistochemistry and immunofluorescence staining

For IHC, paraffin sections were dewaxed, rehydrated, and subjected to antigen retrieval and blocking. Sections were incubated overnight at 4 °C with anti-cytokeratin 14 (CK-14) rabbit monoclonal antibody (1:500; Abcam, Cambridge, UK), followed by incubation with goat anti-rabbit IgG (H+L) secondary antibody (ZSbio, Beijing, China). Sections were developed with DAB and counterstained with hematoxylin. For immunofluorescence staining, sections were incubated overnight at 4 °C with anti-F4/80 rat monoclonal antibody (1:400; Invitrogen) and anti-IL-1β rabbit monoclonal antibody (1:400; Abcam). Secondary antibodies included Alexa Fluor 488-conjugated goat anti-rat IgG and Alexa Fluor 555-conjugated goat anti-rabbit IgG (1:500; Abcam). Images were acquired using an Olympus BX61 upright fluorescence microscope (Olympus, Tokyo, Japan).

### 2.18 Statistical analysis

Statistical analyses were performed using GraphPad Prism 8.0 (GraphPad Software, San Diego, CA, USA). Data were presented as mean ± standard deviation (SD). Normality was assessed using the Shapiro-Wilk test prior to statistical analysis. For comparisons between two groups, an unpaired two-tailed Student’s t-test was used for normally distributed data. For comparisons among multiple groups, one-way analysis of variance (ANOVA) followed by Tukey’s *post hoc* test was used when the data met the assumption of normality. For datasets that did not pass the Shapiro-Wilk normality test, the Kruskal-Wallis test followed by Dunn’s multiple-comparisons test was used instead. Statistical significance was defined as *P < 0.05, **P < 0.01, ***P < 0.001, and ****P < 0.0001; ns, not significant. Results of normality testing were provided in **Supplementary Table 6**. All experiments were performed with at least three independent biological replicates.

## 3 Results

### 3.1 D-mannose reshapes the carbon metabolic network of inflammatory macrophages

**Figure 1:**
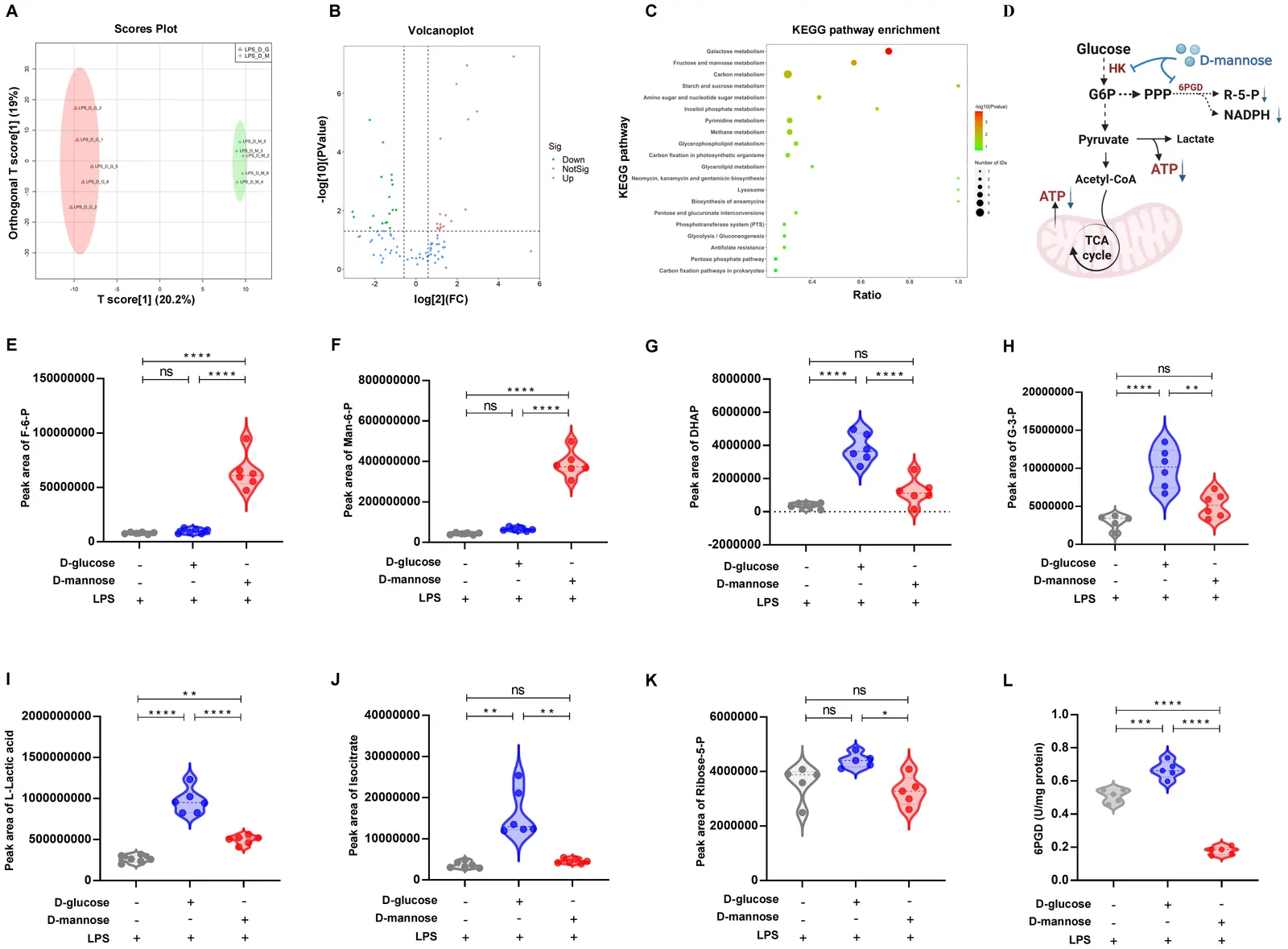
D-mannose reshapes the carbon metabolic network of inflammatory macrophages. **(A–C)** Macrophages were stimulated with LPS (1 μg/mL) and treated with control, 25 mM D-glucose, or 25 mM D-mannose for 24 h, followed by untargeted metabolomics analysis. **(A)** Orthogonal partial least squares discriminant analysis (OPLS-DA) score plot. **(B)** Volcano plot showing the distribution of differential metabolites between the D-glucose and D-mannose groups (upregulated, red; downregulated, green; no significant difference, blue). **(C)** KEGG pathway enrichment analysis of differential metabolites (n = 5). **(D)** Schematic illustration of D-mannose-mediated rewiring of the carbon metabolic network in inflammatory macrophages. HK, hexokinase; 6PGD, 6-phosphogluconate dehydrogenase; PPP, pentose phosphate pathway; TCA, tricarboxylic acid cycle. **(E–K)** Relative abundance of key metabolites in carbon metabolism pathways, represented as peak area: **(E)** fructose-6-phosphate (F-6-P), **(F)** mannose-6-phosphate (Man-6-P), **(G)** dihydroxyacetone phosphate (DHAP), **(H)** glyceraldehyde-3-phosphate (G-3-P), **(I)** L-lactic acid, **(J)** isocitrate, **(K)** ribose-5-phosphate (Ribose-5-P) (n = 5-6). **(L)** 6PGD enzymatic activity, determined by the rate of NADPH generation at 340 nm, in macrophages stimulated with LPS and treated with control, 25 mM D-glucose, or 25 mM D-mannose for 24 h (n = 5). Data are presented as mean ± SD. Normality was assessed using the Shapiro-Wilk test. For normally distributed datasets **(E–I, K, L)**, statistical significance was determined by one-way ANOVA followed by Tukey’s *post hoc* test; for the dataset that did not meet the normality assumption **(J)**, the Kruskal–Wallis test followed by Dunn’s multiple-comparisons test was used. ns, not significant (*P* > 0.05); *P < 0.05, **P < 0.01, ***P < 0.001, ****P < 0.0001.

To investigate the global metabolic impact of D-mannose on inflammatory macrophages, we performed untargeted metabolomics profiling. OPLS-DA revealed a clear spatial separation in overall metabolite profiles between D-mannose- and D-glucose-treated macrophages, indicating a distinct metabolic state transition (**Figure 1A**). A total of 36 differentially abundant metabolites were identified, of which 18 were significantly upregulated and 18 were significantly downregulated (**Figure 1B**). To further elucidate the biological functions of these differential metabolites, we performed KEGG pathway enrichment analysis. “Carbon metabolism” emerged as the significantly enriched pathway upon D-mannose treatment (**Figure 1C**). Carbon metabolism encompasses several core carbon-utilization pathways, such as glycolysis, the pentose phosphate pathway, and the TCA cycle (**Figure 1D**). Specifically, hexose-6-phosphates, including fructose-6-phosphate (F-6-P) and Man-6-P, showed significant accumulation (**Figures 1E, F**). However, the levels of dihydroxyacetone phosphate (DHAP), glyceraldehyde-3-phosphate (G-3-P), and L-Lactic acid, downstream products of glycolytic cleavage, were significantly reduced (**Figures 1G–I**). Metabolic flux entering the mitochondria was similarly restricted, as evidenced by a significant decrease in the key TCA cycle intermediate isocitrate (**Figure 1J**).

The PPP, a major glycolytic branch, was also markedly affected by D-mannose (10). Metabolomics analysis revealed that D-mannose treatment induced a significant accumulation of the PPP metabolite 6-phosphogluconate (6PG), whereas the downstream metabolite ribose-5-phosphate (R5P) was markedly reduced (**Figure 1K**; **Supplementary Figure 1A**). In the oxidative branch of the PPP, 6PGD catalyzes the conversion of 6PG and NADP^+^ to ribulose-5-phosphate and NADPH (14). These findings suggested that D-mannose may inhibit 6PGD enzymatic activity. To validate the metabolomics findings, we measured intracellular 6PGD activity in macrophages stimulated with LPS for 24 hours. Treatment with 25 mM D-mannose significantly suppressed LPS-induced 6PGD activity and reduced the NADPH-generating capacity of macrophages (**Figure 1L**). Collectively, these data demonstrate that D-mannose triggers the accumulation of upstream hexose phosphates while broadly suppressing glycolysis, the TCA cycle, and the PPP, thereby reducing NADPH generation and reshaping the carbon metabolic network of inflammatory macrophages (**Figure 1D**).

### 3.2 D-mannose blocks SAM generation in inflammatory macrophages by suppressing one-carbon metabolism

**Figure 2:**
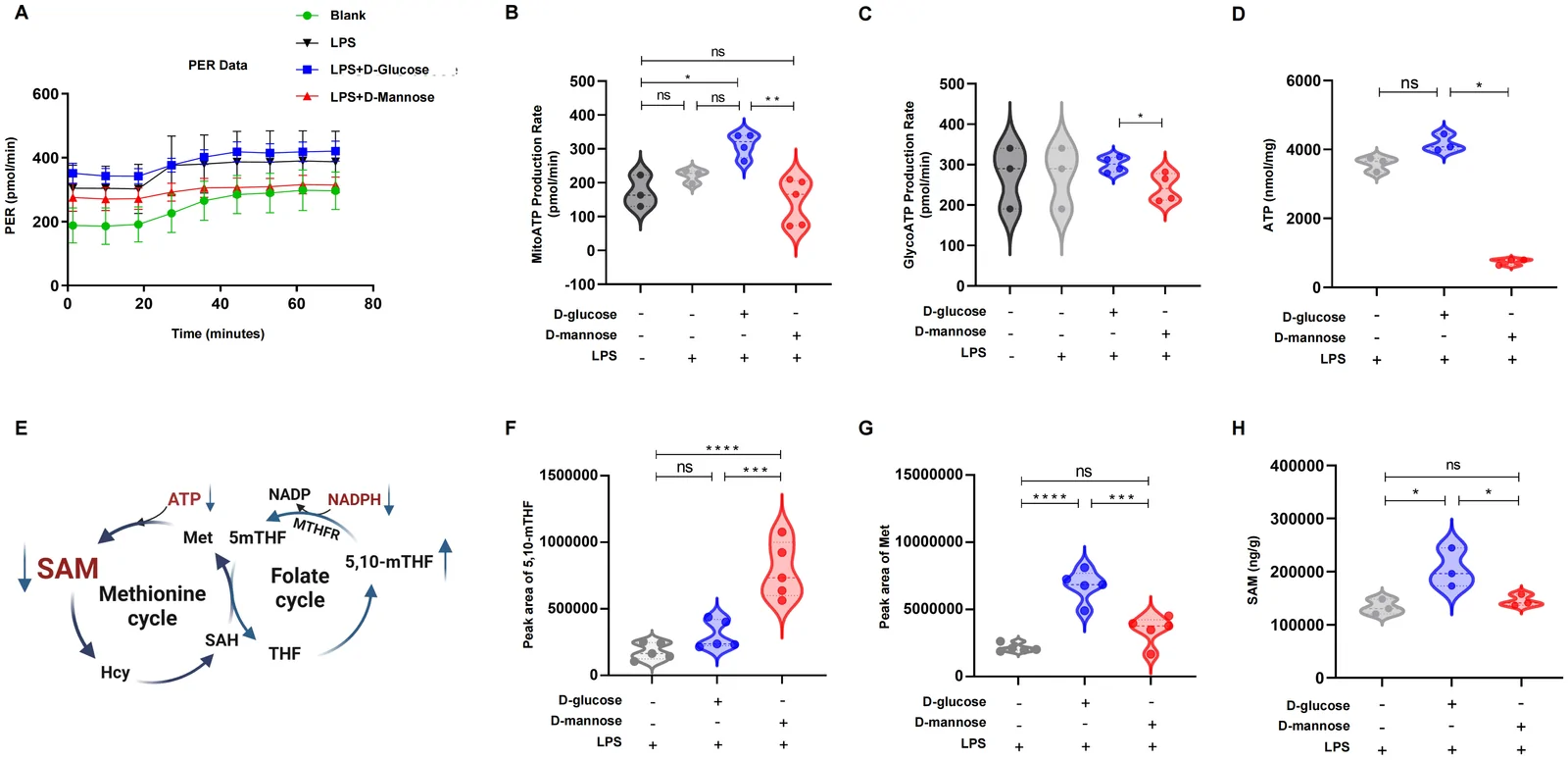
D-mannose blocks SAM generation in inflammatory macrophages by suppressing one-carbon metabolism. **(A–C)** Energy metabolic profiles of macrophages assessed by Seahorse XF Real-Time ATP Rate Assay. **(A)** Real-time proton efflux rate (PER). **(B)** ATP production rate from mitochondrial oxidative phosphorylation (mitoATP) and **(C)** glycolysis (glycoATP), calculated using the Seahorse Report Generator (n = 3–5; mean ± SD). **(D)** Total intracellular ATP levels measured by luciferase-based assay (n = 3; mean ± SD). **(E)** Schematic illustration of D-mannose-mediated inhibition of SAM biosynthesis through one-carbon metabolism, highlighting key metabolites and enzymes: 5,10-mTHF (5,10-methylenetetrahydrofolate), 5-mTHF (5-methyltetrahydrofolate), MTHFR (methylenetetrahydrofolate reductase), Met (methionine), SAM (S-adenosylmethionine). **(F, G)** Relative abundance of key metabolites in one-carbon metabolism and the methionine cycle, represented as peak area: **(F)** 5,10-mTHF, **(G)** methionine (n = 5–6; mean ± SD). **(H)** Intracellular SAM levels measured by ELISA (n = 3; mean ± SD). Normality was assessed using the Shapiro–Wilk test. Statistical significance was determined by one-way ANOVA followed by Tukey’s *post hoc* test for normally distributed datasets **(B, C, F–H)**, whereas the Kruskal–Wallis test followed by Dunn’s multiple-comparisons test was used for the non-normally distributed dataset **(D)**. ns, not significant (*P* > 0.05); *P < 0.05, **P < 0.01, ***P < 0.001, ****P < 0.0001.

Having established that D-mannose inhibits glycolysis and oxidative phosphorylation (OXPHOS) in macrophages, we next investigated how this metabolic suppression impacted cellular energy supply. Using the Seahorse XF Real-Time ATP Rate Assay, we measured OCR and ECAR in LPS-stimulated primary macrophages treated with D-mannose. Compared with 25 mM glucose controls, D-mannose significantly reduced the production rates of both mitochondria-derived ATP (mitoATP) and glycolysis-derived ATP (glycoATP) in LPS-stimulated macrophages (**Figures 2A–C**). Luciferase-based measurement of total cellular ATP levels further confirmed that D-mannose markedly impaired overall ATP supply in LPS-stimulated macrophages (**Figure 2D**).

One-carbon metabolism, comprising the folate and methionine cycles, is highly dependent on energy and reducing equivalents (7, 9) Given the dual suppression of ATP and NADPH production observed in our metabolomics analysis, we examined alterations in the one-carbon metabolic pathway. Metabolomics data revealed that D-mannose induced significant accumulation of the folate cycle intermediate 5,10-mTHF (**Figure 2F**), while downstream components of the methionine cycle were reduced (**Figure 2G**). This phenotype suggests that, due to insufficient ATP and NADPH supply, the MTHFR-mediated conversion of 5,10-mTHF to 5-mTHF was stalled.

Effective one-carbon flux and ATP are essential for the generation of the universal methyl donor SAM (9). We subsequently confirmed that D-mannose treatment significantly suppressed intracellular SAM levels in macrophages (**Figure 2H**). Moreover, concurrent blockade of the PPP and one-carbon metabolism resulted in marked inhibition of downstream *de novo* pyrimidine synthesis (**Supplementary Figures 1B–D**). Collectively, these results demonstrate that D-mannose-induced reductions in ATP and NADPH flux, driven by carbon metabolic suppression, stall one-carbon metabolism and ultimately impair the SAM pool in LPS-stimulated macrophages (**Figure 2E**).

### 3.3 D-mannose epigenetically represses IL-1β by suppressing SAM synthesis

**Figure 3:**
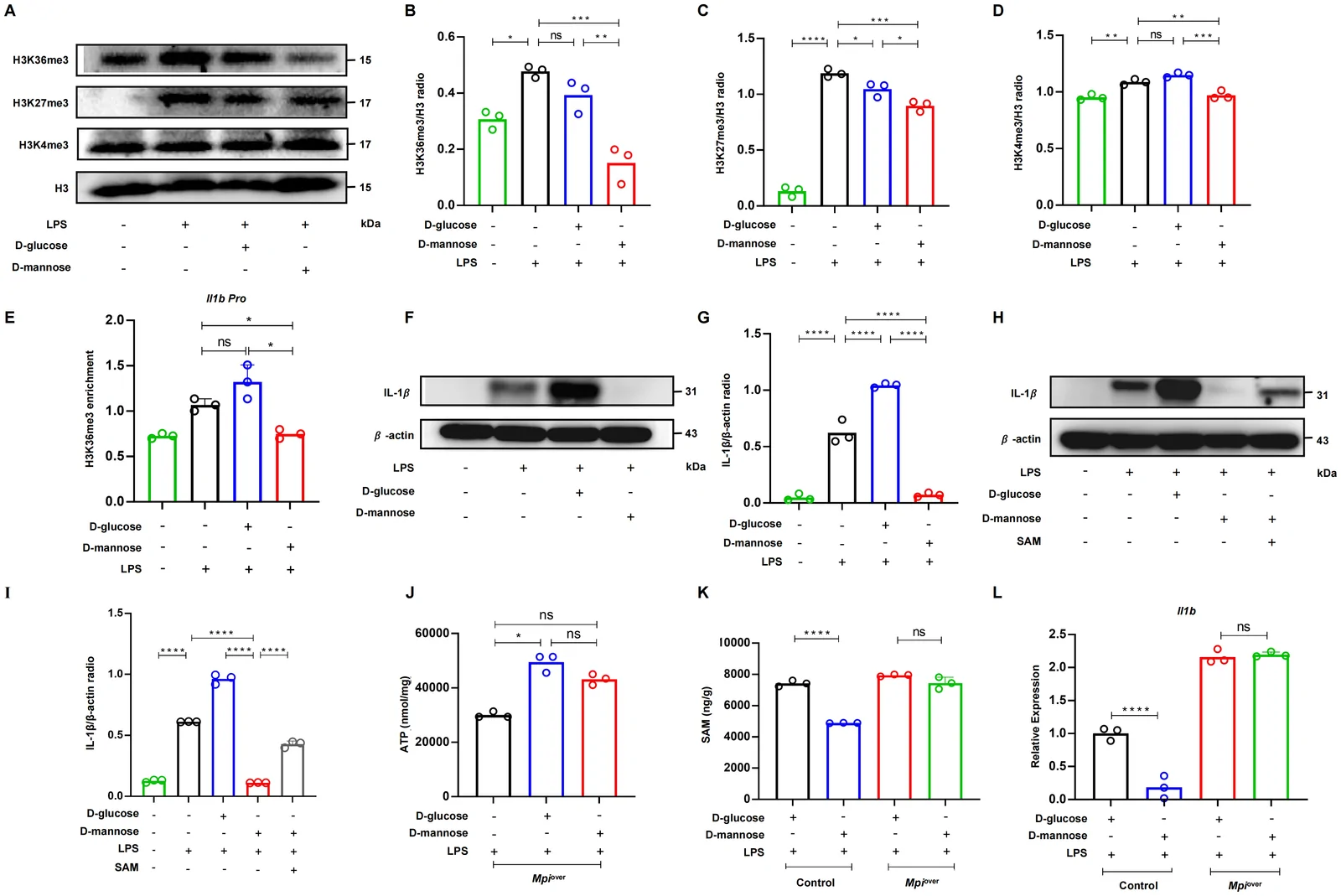
D-mannose epigenetically represses IL-1β by suppressing SAM synthesis. **(A)** Western blot analysis of histone methylation levels (H3K36me3, H3K27me3, H3K4me3) in macrophages stimulated with LPS (1 μg/mL) and treated with control, 25 mM D-glucose, or 25 mM D-mannose for 24 h. Histone H3 served as the loading control. **(B–D)** Densitometric analysis of H3K36me3, H3K27me3, and H3K4me3 protein levels normalized to Histone H3 and presented as normalized band intensities (n=3; mean ± SD). **(E)** ChIP analysis of H3K36me3 enrichment at the *Il1b* promoter region (n=3; mean ± SD). **(F)** Western blot analysis of IL-1β protein expression in macrophages. β-actin served as the loading control. **(G)** Densitometric analysis of IL-1β protein levels normalized to β-actin and presented as normalized band intensities (n=3; mean ± SD). **(H)** Western blot analysis of IL-1β protein expression following exogenous SAM supplementation. β-actin served as the loading control. **(I)** Densitometric analysis of IL-1β protein levels normalized to β-actin and presented as normalized band intensities (n=3; mean ± SD). **(J)** Total intracellular ATP levels in macrophages with *Mpi* overexpression measured by luciferase-based assay (n=3; mean ± SD). **(K)** Intracellular SAM levels were measured by ELISA following *Mpi* overexpression (n = 3, mean ± SD). **(L)** Real-time PCR analysis of *Il1b* transcription levels following *Mpi* overexpression (n=3; mean ± SD). Normality was assessed using the Shapiro–Wilk test. Statistical significance was determined by one-way ANOVA followed by Tukey’s *post hoc* test for normally distributed datasets **(B–E, G, I, K, L)**, whereas the Kruskal–Wallis test followed by Dunn’s multiple-comparisons test was used for the non-normally distributed dataset **(J)**. ns, not significant (*P* > 0.05); *P < 0.05, **P < 0.01, ***P < 0.001, ****P < 0.0001.

SAM is a critical methyl donor that drives epigenetic reprogramming (15). We therefore hypothesized that D-mannose-induced SAM impairment may further remodel the chromatin landscape of macrophages. To test this, we examined several key histone H3 methylation marks associated with transcriptional accessibility by Western blot (6). Compared with the D-glucose control group, D-mannose significantly suppressed histone methylation, including H3K36me3, H3K27me3, and H3K4me3. Among these modifications, the most prominent reduction was observed for H3K36me3, an activating histone modification (**Figures 3A–D**). We next investigated the impact of D-mannose on the inflammatory transcriptional program of macrophages by real-time PCR. D-mannose (25 mM) significantly suppressed LPS-induced expression of pro-inflammatory cytokine genes (*Il1b*, *Il6*, and *Tnfa*), with the most pronounced inhibitory effect observed on *Il1b* transcription (**Supplementary Figures 2A–D**). In contrast, D-mannose exerted no significant effect on IL-4-induced anti-inflammatory genes (*Tgfb*, *Arg1*, and *Il10*) (**Supplementary Figure 2E**). Given that *Il1b* was the most robustly suppressed target, we performed ChIP analysis to delineate the epigenetic dynamics at this specific locus. D-mannose treatment significantly diminished LPS-induced H3K36me3 enrichment at the *Il1b* promoter (**Figure 3E**). This loss of the activating epigenetic mark at the target gene promoter was consistent with the marked downregulation of IL-1β protein expression detected by Western blot (**Figures 3F, G**).

These findings suggested that D-mannose-mediated epigenetic and transcriptional suppression of IL-1β may be attributable to restricted intracellular SAM availability. To test this causal relationship, we supplemented the D-mannose-containing culture system with 0.1 mM exogenous SAM. SAM supplementation reversed the D-mannose-imposed suppression of H3K36me3 enrichment at the *Il1b* promoter and *Il1b* expression (**Figures 3H, I**; **Supplementary Figures 2F, G**). This finding establishes SAM as the critical mediator linking D-mannose-induced metabolic perturbation to downstream inflammatory gene repression. Due to the inherently low basal *Mpi* expression in macrophages—a “metabolic bottleneck”—Man-6-P generated from D-mannose metabolism tends to accumulate intracellularly, feedback-inhibiting the activity of HK and triggering metabolic arrest (10, 12). To determine whether D-mannose elicits this cascade of downstream suppression through MPI-limited Man-6-P accumulation, we generated *Mpi*-overexpressing macrophages using lentiviral transduction (**Supplementary Figure 2H**). Metabolic rescue analysis revealed that *Mpi* overexpression attenuated the D-mannose-mediated suppression of LPS-induced ATP production (**Figure 3J**). In contrast, in non-transduced macrophages, D-mannose reduced ATP production by 82.0% (**Figure 2D**). More critically, accompanying the restoration of energy metabolic capacity, *Mpi* overexpression abolished the inhibitory effect of D-mannose on SAM production and IL-1β expression (**Figures 3K, L**). These results systematically delineate a mechanism by which D-mannose reshapes the macrophage transcriptional state through altering its metabolic state. Mechanistically, D-mannose imposes a blockade on carbon metabolism, thereby disrupting one-carbon metabolism-driven SAM synthesis. The resulting SAM deficit reduces H3K36me3 enrichment at the *Il1b* promoter, ultimately attenuating *Il1b* expression (Graphical Abstract).

### 3.4 Topical application of D-mannose significantly accelerates full-thickness skin wound healing in mice

**Figure 4:**
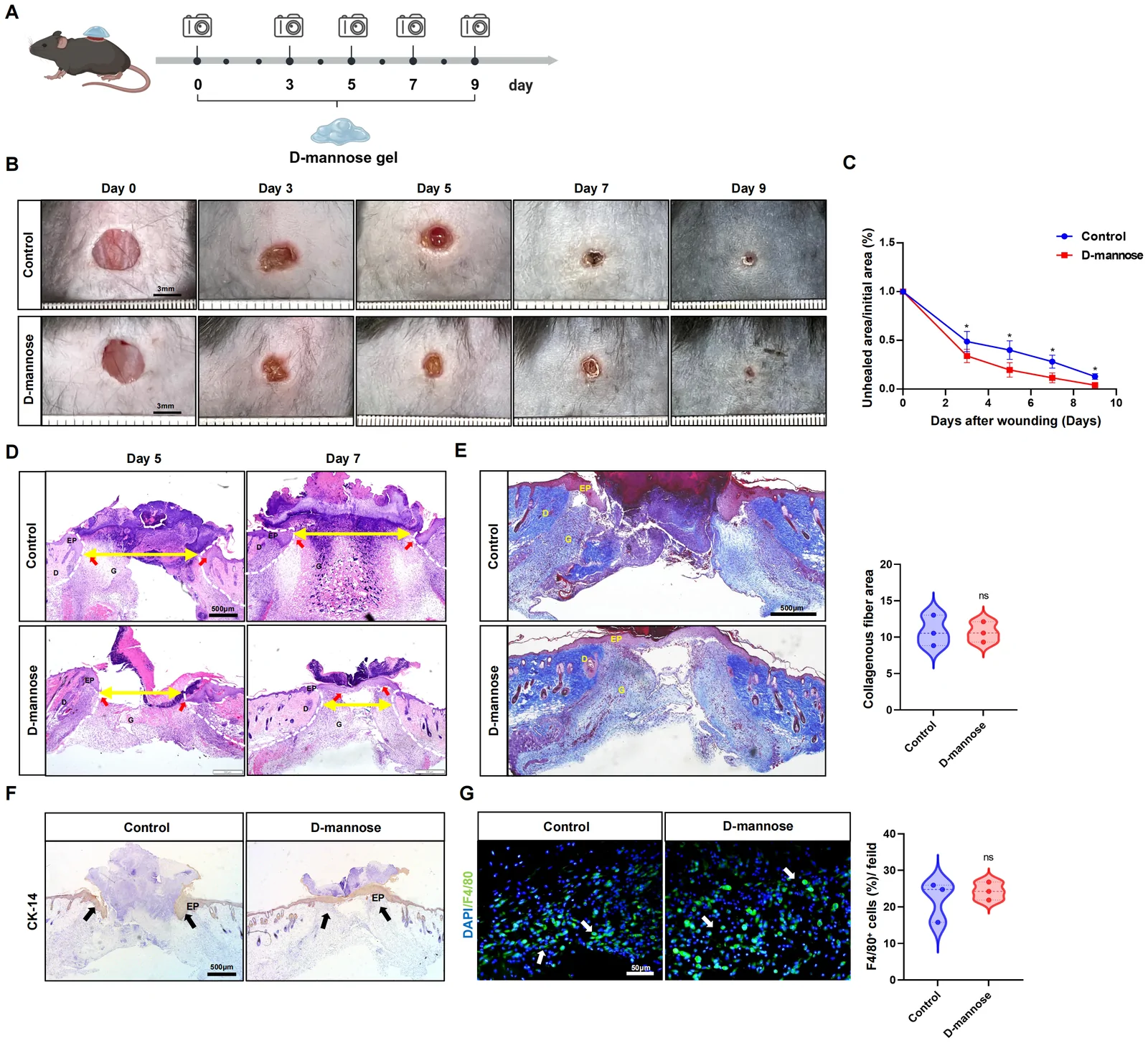
Topical application of D-mannose significantly accelerates full-thickness skin wound healing in mice. **(A)** Experimental workflow: full-thickness excisional wounds were created on the dorsal skin of male C57BL/6J mice aged 8–10 weeks. **(B)** Representative macroscopic images of wounds in control and D-mannose groups at days 0, 3, 5, 7, and 9 post-wounding. **(C)** Wound healing kinetics. Relative wound unhealed rates at different time points were quantified and compared between control and D-mannose groups (n = 7; mean ± SD). **(D)** H&E staining of wound tissues. Wound edges (red arrows) and wound diameter (yellow lines) were assessed at days 5 and 7 post-wounding to evaluate re-epithelialization and wound contraction. **(E)** Masson’s trichrome staining of wound tissues. Collagen deposition at day 7 post-wounding was quantified using ImageJ (n=3; mean ± SD). **(F)** Immunohistochemical staining of CK-14 in wound tissues. Re-epithelialization areas (black arrows) were observed to assess epithelialization. **(G)** Immunofluorescence staining of F4/80 in wound tissues. F4/80-positive macrophage infiltration (green fluorescence) was quantified; DAPI labeled nuclei (blue fluorescence) (n=3; mean ± SD). Normality was assessed using the Shapiro–Wilk test. Statistical significance was determined by one-way ANOVA followed by Tukey’s *post hoc* test for normally distributed datasets **(C, E, G)**. Scale bars: **(D–F)** 500 μm; **(G)** 50 μm. ns, not significant (*P* > 0.05); *P < 0.05.

Based on the mechanism elucidated above, we next explored the therapeutic potential of this strategy as a metabolism-targeted intervention for inflammatory tissue injury *in vivo*. To determine the effect of D-mannose on wound healing, we established a full-thickness dorsal skin wound model in mice and topically applied either 1.1 M D-mannose gel or PBS gel (**Figure 4A**). Wound area measurements at various time points post-injury revealed that topical application of D-mannose significantly accelerated dorsal wound closure in mice (**Figures 4B, C**). H&E staining and CK-14 immunohistochemistry further confirmed that D-mannose treatment markedly promoted wound contraction and re-epithelialization (**Figures 4D, F**). Quantitative assessment of local collagen deposition by Masson’s trichrome staining revealed no significant differences in collagen fiber formation among the experimental groups (**Figure 4E**). Given the critical regulatory role of macrophages in wound healing, we next examined macrophage infiltration within wound tissues by immunofluorescence staining. Immunofluorescence staining for the macrophage surface marker F4/80, followed by quantification of F4/80^+^ cells using ImageJ, revealed abundant macrophage infiltration in wound tissues across all experimental groups during the early phase of wound healing (day 3 post-injury), with no significant differences among groups (**Figure 4G**). Collectively, these results support that D-mannose significantly promotes wound healing and facilitates wound contraction and re-epithelialization.

### 3.5 D-mannose promotes wound healing by suppressing macrophage IL-1β expression

**Figure 5:**
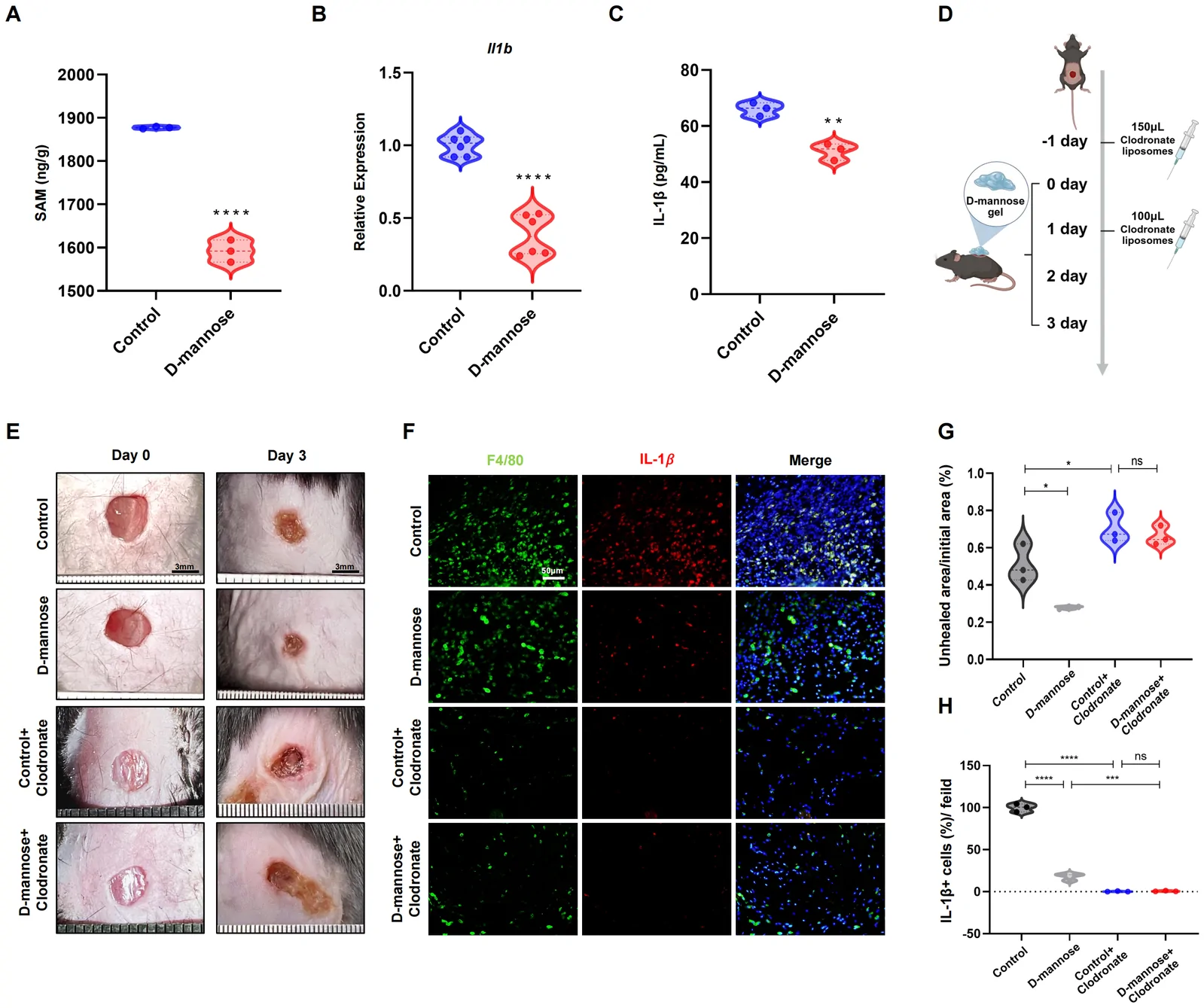
D-mannose promotes wound healing by suppressing macrophage IL-1β expression. **(A)** Intrawound SAM levels in control and D-mannose groups measured by ELISA (n = 3; mean ± SD). **(B)** Real-time PCR analysis of *Il1b* mRNA expression in wound tissues from control and D-mannose groups (n = 6; mean ± SD). **(C)** Serum IL-1β levels in control and D-mannose groups measured by ELISA (n = 3; mean ± SD). **(D)** Experimental workflow: male C57BL/6J mice aged 8–10 weeks received intraperitoneal injection of clodronate liposomes for macrophage depletion, followed by creation of full-thickness excisional wounds on the dorsal skin. **(E)** Representative macroscopic images of skin wounds at days 0 and 3 post-wounding in control, D-mannose, control + clodronate liposomes, and D-mannose + clodronate liposomes groups. **(F)** Immunofluorescence co-staining of F4/80 and IL-1β in wound tissue sections at day 3 post-wounding. Green fluorescence indicates F4/80-positive cells; red fluorescence indicates IL-1β-positive cells; yellow fluorescence indicates co-localization; blue fluorescence (DAPI) indicates nuclei. **(G)** Relative wound unhealed rates at day 3 post-wounding were measured using ImageJ (n=3; mean ± SD). **(H)** Quantification of relative IL-1β fluorescence intensity from the immunofluorescence staining in **(F)** (n=3; mean ± SD). Normality was assessed using the Shapiro–Wilk test. Statistical significance was determined by one-way ANOVA followed by Tukey’s *post hoc* test for normally distributed datasets **(A–C, G, H)**. Scale bar = 50 μm in **(F)**. ns, not significant (*P* > 0.05); *P < 0.05, **P < 0.01, ***P < 0.001, ****P < 0.0001.

To determine whether the *in vivo* anti-inflammatory and pro-healing effects of D-mannose recapitulate the mechanisms observed *in vitro*, we assessed local and systemic metabolic and inflammatory profiles at day 3 post-injury. Metabolite quantification and real-time PCR analysis revealed that topical application of D-mannose not only significantly reduced SAM levels in wound tissues but also downregulated the mRNA expression of pro-inflammatory mediators (*Il1b*, *Il6*, *Tnfa*, and *Nos2*) at the wound site (**Figures 5A, B**; **Supplementary Figure 3A**). Consistent with these findings, ELISA confirmed a significant decrease in IL-1β, IL-6, and TNF-α protein levels within wound tissues (**Supplementary Figure 3B**). Serological analysis further demonstrated that topical D-mannose application not only ameliorated the local inflammatory microenvironment but also significantly reduced circulating IL-1β levels in the systemic circulation (**Figure 5C**). In contrast, during the later phase of wound repair (day 7 post-injury), D-mannose exerted no significant effect on the expression of anti-inflammatory and tissue-remodeling genes (*Tgfb*, *Arg1*, and *Il10*) (**Supplementary Figure 3C**). To determine whether the anti-inflammatory and pro-healing effects of D-mannose are macrophage-dependent, we established a macrophage depletion model using clodronate liposomes (**Figure 5D**). Macrophage depletion completely abolished the wound repair benefits conferred by D-mannose (**Figures 5E, G**). Immunofluorescence co-localization further confirmed that F4/80 and IL-1β co-localized within the wound microenvironment, and that macrophage depletion nearly eliminated local IL-1β expression. D-mannose treatment significantly reduced the proportion of F4/80^+^IL-1β^+^ macrophages (**Figures 5F, H**). Together, these data validate that D-mannose, acting as a metabolism-targeted intervention, restricts local SAM abundance *in vivo*, reshapes the inflammatory secretory phenotype of macrophages, and thereby drives accelerated wound healing.

## 4 Discussion

Chronic wounds and non-healing wounds remain significant challenges in clinical wound management, often hampered by a prolonged inflammatory phase (16). Persistent macrophage activation is considered a key driver of this pathological inflammation (17). The present study demonstrates that D-mannose restricts one-carbon metabolism and attenuates macrophage inflammatory function through a metabolism–epigenetic axis. Topical D-mannose supplementation ameliorates the wound inflammatory microenvironment and accelerates wound contraction and re-epithelialization, representing a viable metabolism-targeted intervention strategy.

One-carbon metabolism provides critical metabolic support for immune cell effector functions (18–20). This network is centered on the folate and methionine cycles (21). The folate cycle supplies one-carbon units to support the methionine cycle as well as *de novo* purine and thymidine synthesis (22, 23). The methionine cycle sustains methylation reactions that participate in epigenetic regulation, including DNA and histone methylation, and is intimately linked to redox homeostasis (24). In inflammatory macrophages, one-carbon metabolism has been reported to enhance IL-1β production by supporting SAM and glutathione biosynthesis (6, 25). Our data reveal that, D-mannose markedly restricts the carbon metabolic network of inflammatory macrophages, with diminished flux through glycolysis, the TCA cycle, and the PPP. The concurrent reduction in ATP and NADPH generation stalls one-carbon metabolism and depletes the SAM pool, thereby suppressing SAM-dependent pro-inflammatory transcriptional programs.

In inflammatory macrophages, metabolites can serve as substrates or cofactors for epigenetic enzymes, thereby coupling metabolic reprogramming to epigenetic modifications; notable examples include ATP, acetyl-CoA, lactate, and SAM (26–29). We observed that D-mannose-induced SAM impairment diminished the activating H3K36me3 mark at the *Il1b* promoter upon LPS stimulation, resulting in epigenetic repression of IL-1β. Intriguingly, this metabolite-driven epigenetic remodeling exhibits marked cell type specificity. A recent study demonstrated that, in CD8^+^ memory T cells, D-mannose—despite suppressing glycolysis—enhances TCA cycle activity to promote α-ketoglutarate (α-KG) accumulation, which in turn modulates H3K4 methylation to maintain epigenetic stemness (30). In contrast, the present study reveals that in macrophages, the metabolic restriction imposed by D-mannose along the “carbon metabolism–one-carbon metabolism” axis directly reduces the core methyl donor SAM, thereby exerting transcriptional suppression at the epigenetic level.

## 5 Conclusions

In summary, this study elucidates the mechanism by which D-mannose diminishes *Il1b* expression in inflammatory macrophages through metabolic and epigenetic reprogramming. Topical application of D-mannose significantly ameliorates the wound inflammatory microenvironment, promotes wound contraction and re-epithelialization, and accelerates full-thickness skin wound healing. These findings provide a mechanistic foundation for D-mannose as a metabolism-targeted epigenetic intervention in inflammatory wound repair.

## Data Availability

The original contributions presented in the study are included in the article/**Supplementary Material**. Further inquiries can be directed to the corresponding authors.
