# Supplementary material for: D-mannose blocks S-adenosylmethionine generation to suppress macrophage IL-1β expression
Source: Front Immunol. 2026 Aug 21;17:1895029. doi: 10.3389/fimmu.2026.1895029 (PMC13541704; doi:10.3389/fimmu.2026.1895029)
Supplement: Supplementary file 1 [file DataSheet1.docx]

**Supplementary Figure 1**. (A–D) Macrophages were stimulated with 1 μg/mL LPS and treated with control, 25 mM D-glucose, or 25 mM D-mannose for 24 h, followed by untargeted metabolomics analysis. Relative abundance of key metabolites in the pyrimidine metabolism pathway, represented as peak area: (A) 6-phosphogluconic acid (6PG), (B) cytidine, (C) deoxythymidine monophosphate (dTMP), (D) dihydrouracil (n = 5–6; mean ± SD). Statistical significance was determined by one-way ANOVA followed by Tukey’s post hoc test. ns, not significant (P > 0.05); *P < 0.05, **P < 0.01, ***P < 0.001, ****P < 0.0001.

**Supplementary Figure 2.** (A) Real-time PCR analysis of *Il1b*, *Il6*, and *Tnfa* expression in macrophages stimulated with LPS and treated with control, 25 mM D-glucose, or 25 mM D-mannose for 24 h (n = 3-6; mean ± SD). (B) IL-1β levels in macrophage culture supernatants measured by ELISA following LPS stimulation with control, 25 mM D-glucose, or 25 mM D-mannose treatment for 24 h (n = 4; mean ± SD). (C) Flow cytometry analysis of the proportion of pro-IL-1β⁺ macrophages following LPS stimulation with control, 25 mM D-glucose, or 25 mM D-mannose treatment for 24 h. (D) Immunofluorescence staining of IL-1β on cell coverslips after 1 μg/mL LPS stimulation for 24 h. Red fluorescence indicates IL-1β-positive cells; blue fluorescence (DAPI) indicates nuclei (scale bar = 25 μm). (E) Real-time PCR analysis of *Tgfb*, *Arg*1, and *Il10* expression in macrophages induced with 100 ng/mL IL-4 and treated with control, 25 mM D-glucose, or 25 mM D-mannose for 24 h (n = 3–6; mean ± SD). (F) ChIP analysis of H3K36me3 enrichment at the *Il1b* promoter region (n=3; mean ± SD). (G) IL-1β secretion levels in macrophage culture supernatants measured by ELISA (n=3; mean ± SD). (H) Real-time PCR confirming significant upregulation of *Mpi* expression in macrophages following lentiviral transduction (n=3; mean ± SD). Statistical significance was assessed by one-way ANOVA followed by Tukey’s post hoc test in (A-C, E, F and G), and by Student’s t-test in (H). ns, not significant (P > 0.05); *P < 0.05, **P < 0.01, ***P < 0.001, ****P < 0.0001.

**Supplementary Figure 3.** (A) Real-time PCR analysis of *Il6*, *Tnfa*, and *Nos2* expression in wound tissues (day 3 post-wounding) from control and D-mannose groups (n = 6; mean ± SD). (B) Intrawound IL-1β, IL-6, and TNF-α protein levels in control and D-mannose groups measured by ELISA (n = 3–4; mean ± SD). (C) Real-time PCR analysis of *Tgfb*, *Il10*, and *Arg1* expression in wound tissues (day 7 post-wounding) from control and D-mannose groups (n = 3–6; mean ± SD). Statistical significance was determined by Student’s t-test. ns, not significant (P > 0.05); *P < 0.05, **P < 0.01, ***P < 0.001, ****P < 0.0001.
